# Stress-Induced MazF-Mediated Proteins in Escherichia coli

**DOI:** 10.1128/mBio.00340-19

**Published:** 2019-03-26

**Authors:** Akanksha Nigam, Tamar Ziv, Adi Oron-Gottesman, Hanna Engelberg-Kulka

**Affiliations:** a Department of Microbiology and Molecular Genetics, IMRIC, The Hebrew University Hadassah Medical School, Jerusalem, Israel; b Smoler Proteomics Center, Faculty of Biology, Technion, Haifa, Israel; Harvard University; University of Minnesota Medical School; University of Southern California

**Keywords:** bacterial stress response, toxin-antitoxin, proteomics

## Abstract

Escherichia coli *mazEF* is an extensively studied stress-induced toxin-antitoxin (TA) system. The toxin MazF is an endoribonuclease that cleaves RNAs at ACA sites. By that means, under stress, the induced MazF generates a stress-induced translation machinery (STM) composed of MazF-processed mRNAs and selective ribosomes that specifically translate the processed mRNAs. Here, we performed a proteomic analysis of all the *E. coli* stress-induced proteins that are mediated through the chromosomally borne *mazF* gene. We show that the mRNAs of almost all of them are characterized by the presence of an ACA site up to 100 nucleotides upstream of the AUG initiator. Therefore, under stressful conditions, induced MazF processes mRNAs that are translated by STM. Furthermore, the presence of the ACA sites far upstream (up to 100 nucleotides) of the AUG initiator may still permit translation by the canonical translation machinery. Thus, such dual-translation mechanisms enable the bacterium under stress also to prepare proteins for immediate functions while coming back to normal growth conditions.

## Abstract (precis)

The stress response, the strategy that bacteria have developed in order to cope up with all kinds of adverse conditions, is so far understood at the level of transcription. Our previous findings of a uniquely modified stress-induced translation machinery (STM) generated in E. coli under stress by the endoribonucleolytic activity of the toxin MazF opens a new chapter in understanding microbial physiology under stress at the translational level. Here, we performed a proteomic analysis of all the E. coli stress-induced proteins that are mediated by chromosomally borne MazF through STM.

## INTRODUCTION

Toxin-antitoxin (TA) systems are abundant on the chromosomes of most bacteria. Among these, the first one discovered and the one most studied is Escherichia coli *mazEF* (1). *mazF* specifies for the stable toxin MazF, and *mazE* specifies for the labile antitoxin MazE that interferes with MazF toxicity. MazE is degraded by protease ClpPA (1). E. coli *mazEF* is triggered by various stressful conditions, including DNA damage caused by treatment with nalidixic acid (NA) (2, 3).

E. coli MazF was first described as a sequence-specific mRNA endoribonuclease (designated mRNA interferase) that preferentially cleaves mRNAs at ACA sites, thereby inhibiting protein synthesis (4). Subsequently, we found that E. coli MazF also targets the 16S rRNA within the 30S ribosomal subunit at the decoding center, thereby removing 43 nucleotides from the 3′ terminus (5). The region removed includes the anti-Shine-Dalgarno (anti-SD) region, resulting in the production of deficient ribosomes. Thus, under stressful conditions, when MazF is induced, special translation machinery, the STM system, is generated. It is composed of (i) MazF, which generates mRNAs by cleaving at ACA sites immediately adjacent to or upstream from the AUG start codons of specific mRNAs (5, 6), and (ii) deficient ribosomes that are selectively able to translate the processed mRNAs (5, 7). Thus, under stress, the induction of MazF leads to the generation of a novel MazF regulon that is translated by the novel stress ribosomes, resulting in the production of stress proteins.

Pulsed stable-isotope labeling in tissue culture (SILAC) was used in several studies for identifications of newly synthesized proteins (8–11) but rarely in bacterial systems (12). Here, we used this technique to perform a proteomic analysis of the stress-induced proteins whose translation was mediated by chromosomally borne *mazF* in E. coli.

## RESULTS

### Characterization of E. coli proteins induced by a DNA-damaging agent and mediated by chromosomally borne *mazEF*.

**FIG 1:**
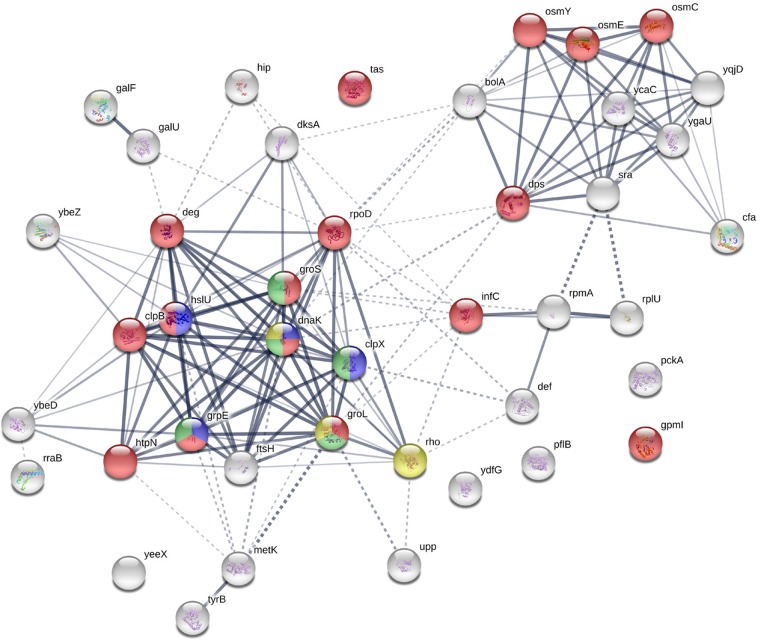
Interaction map and annotation enrichment of the newly synthesized *mazEF*-related proteins. String software was used to analyze the interactions. The nodes are colors based on the enriched annotations as detailed here. For Gene Ontology Biological Process (GOBP) identifier (ID) 0043335, the pathway was protein unfolding, the gene count was 4, the FDR was 3.19e–05, and the color is blue. For GOBP ID 0006950, the pathway was response to stress, the gene count was 16, the FDR was 0.00567, and the color is red. For GOBP ID 0019538, the pathway was protein metabolic process, the gene count was 14, the FDR was 0.015, and the color is green. For GOBP ID 0006457, the pathway was protein folding, the gene count was 5, the FDR was 0.0324, and the color is light green. For KEGG ID 03018, the pathway was RNA degradation, the gene count was 3, the FDR was 0.0445, and the color is yellow. For InterPro ID IPR003959, the pathway was ATPase with the AAA-type core, the gene count was 5, the FDR was 9.23e–06, and the color is pink.

In our experiments, we used E. coli strain MC4100 *relA*^+^ and compared it to its Δ*mazEF* deletion derivative. To damage the DNA, we used nalidixic acid (NA), which, in previous work we found to be an efficient inducer of *mazF* (2). To conduct our proteomic analysis, we used the pulsed SILAC approach. In those experiments, heavy (H) lysine and arginine (containing heavy isotopes of carbon and nitrogen) were added to the medium for a short time. Proteins synthesized after exposure to heavy amino acids contained heavy lysine and arginine, whereas proteins synthesized prior to that time contained only light (L) variants. We determined the relative amounts of newly synthetized proteins in the mutant and wild-type (WT) samples following NA treatment by comparing a naturally occurring light isotope and a heavy new protein (H/L ratio). Previous studies done in vertebrate cells were analyzed after several hours. In our bacterial system, we checked several time points and deduced that 5 min is the optimal time point to figure out which are the differential new proteins. Several (1,949) proteins were identified, and in 832 of them, newly synthetized heavy proteins were detected in one of the samples. We repeated the experiments 3 times and looked for proteins that were induced more in the WT than in the *ΔmazEF* mutants in all the repeats (see Fig. S1 in the supplemental material). Table 1 represents the list of proteins that were induced by the NA treatment in the WT. The list of proteins induced by NA treatment was obtained by comparing the H/L values from all the WT samples to those of the mutant samples. We obtained a total of 42 proteins, many of which are known to participate in the stress response (Fig. 1; see also Discussion). Remarkably, with the exception of six of them, in all the corresponding mRNAs specifying these proteins, the MazF cleavage site ACA was found to be located up to 100 nucleotides upstream of the AUG initiator. It seems that the presence of these ACAs upstream of the AUG initiator is important for the expression of the described stress-induced MazF-mediated proteins. We also carried bioinformatics studies of all E. coli genes that carry an ACA site to 100 nucleotides upstream of the AUG initiator. We found that 2,807 genes encode a free region upstream. Among them, 2,229 have an ACA site to 100 nucleotides. Since in our proteomic study only 42 proteins were detected (Table 1), we assume that the existence of an ACA site upstream of the initiation codon is necessary but not enough for the synthesis of the stress-induced MazF-mediated proteins *in* E. coli. Alternatively, the proteins may simply not have been expressed or were degraded under the conditions being studied.

**TABLE 1:** Proteins induced in E. coli by chromosomally borne *mazF* after nalidixic acid treatment^a^

Gene name	Protein product	Relative intensity of new protein increment or comment	Possible cutting site	Distance of the possible cutting sites from the AUG initiator (nt)
*bolA*	DNA-binding transcriptional regulator	2.22	ACA	29
*cfa*	Cyclopropane-fatty-acyl-phospholipid synthase	1.4	ACU	2
*clpB*	Chaperone protein ClpB	3.3	ACA	86
*clpX*	ATP-dependent Clp protease ATP-binding subunit ClpX	1.69	ACA	20
*def*	Peptide deformylase	1.28	ACA	15
*dksA*	RNA polymerase binding transcription factor	1.95	ACA	1
*dnaK*	Chaperone protein	3.66	ACA	33
*dps*	DNA protection during starvation protein	2.32	ACA	20
*ftsH*	ATP-dependent zinc metalloprotease FtsH	9.96	ACA	1
*galF*	UTP-glucose-1-phosphate uridylyltransferase	1.52	ACA	15
*galU*	UTP-glucose-1-phosphate uridylyltransferase	1.46	ACA	18
*gpml*	2,3-Bisphosphoglycerate-independent phosphoglycerate mutase	1.29	ACU	15
*groL*	60-kDa chaperonin	3.46	ACA	25
*groS*	10-kDa chaperonin	2.46	ACA	32
*grpE*	Protein GrpE	2.09	ACA	90
*hslU*	ATP-dependent protease ATPase subunit	2.41	ACA	19
*ibpA*	Small heat shock protein	1.86	ACA	30
*ihfB*	Integration host factor	1.60	ACA	40
*infC*	Translation initiation factor IF-3	1.36	ACA	18
*lon*	Lon protease	5.8	ACA	50
*metK*	*S-*Adenosylmethionine synthase	1.64	ACA	40
*osmC*	Peroxiredoxin	1.7	ACA	2
*osmE*	Osmotically inducible lipoprotein E	4.58	ACA	14
*osmY*	Osmotically inducible protein Y	2.78	ACA	13
*pckA*	Phosphoenolpyruvate carboxykinase	3.79	ACA	23
*pflB*	Formate acetyltransferase 1	1.76	ACA	1
*rho*	Transcription termination factor	1.40	ACA	7
*rplU*	50 S ribosomal protein	1.57	ACU	42
*rpmA*	50 S ribosomal protein	1.43	ACA	89
*rpoD*	RNA polymerase sigma factor	1.92	ACA	35
*rraB*	Regulator of ribonuclease activity	1.79	ACA	18
*sra*	Stationary-phase-induced ribosome-associated protein	1.55	ACA	18
*tas*	Protein Tas	1.44	ACG	5
*tyrB*	Aromatic-amino-acid aminotransferase	8.6	ACA	74
*upp*	Uracil phosphoribosyl transferase	Expressed only in MC4100 *relA^+^*	ACG	81
*ybeD*	Uncharacterized protein	3.84	ACA	1
*ybeZ*	PhoH-like protein	1.94	ACA	17
*ycaC*	Uncharacterized protein	Expressed only in MC4100 *relA^+^*	ACA	93
*ydfG*	NADP-dependent 3-hydroxy acid dehydrogenase	1.83	ACA	7
*yeeX*	Uncharacterized protein	1.43	ACA	19
*ygaU*	Uncharacterized protein	2.27	ACA	37
*yqjD*	Uncharacterized protein	1.66	ACU	2

a E. coli strain MC4100 *relA*^+^ and its *ΔmazEF* derivative were grown to mid-logarithmic phase (OD_600_, 0.5). Both cultures were treated with 100 µg/ml of nalidixic acid (NA) for 10 min at 37°C without shaking. The heavy amino acids arginine (_15_N^4^) and lysine (_15_N^2^) were added to both the NA-induced cultures at a concentration of 100 µg/ml and further incubated at 37°C for 5 min. Cells were centrifuged and sent for proteomic analysis. The table demonstrates the relative intensities of new protein increment in the H/L ratio of MC4100 *relA*^+^ divided by the H/L ratio of its *ΔmazEF* derivative. The data represent the means of results from triplicate experiments. The last column shows the numbers of nucleotides (distance) from the possible cutting sites to the AUG initiator of each mRNA. nt, nucleotides.

10.1128/mBio.00340-19.1 FIG S1 Scatter plot correlating the H/L values of the WT sample to those of the mutant sample. The Perseus software was used to visualize the correlation between the H/L values of the WT sample to those of the mutant sample. Examples of a few differential proteins that can be found in Table 1 are labeled in red. Download FIG S1, TIF file, 0.01 MB. Copyright © 2019 Nigam et al. 2019 Nigam et al. This content is distributed under the terms of the Creative Commons Attribution 4.0 International license.

### The distance of an ACA site upstream of the AUG initiator is important for the expression of a stress-induced MazF-mediated protein.

**FIG 2:**
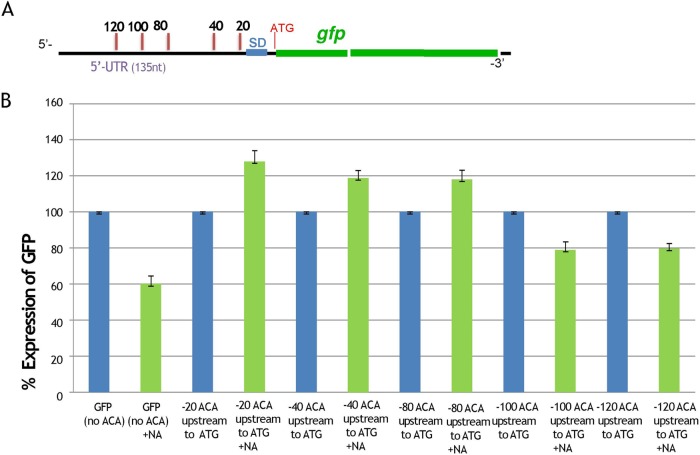
The distance of the ACA site from the AUG initiation codon plays a role in the expression of the stress-induced GFP reporter. (A) GFP reporter system for studying the effect of the location of ACA sites upstream from the AUG initiation codon on translation under stress. We used a *gfp* reporter gene that carries no ACA sites in its coding sequence and no ACA sites upstream (up to 135 nucleotides) from the ATG initiation codon (13). We used this reporter system as a basis for generating five new constructs. In each new construct, we inserted an ACA site upstream from the ATG initiation codon at specific locations, nucleotides 20, 40, 80, 100, and 120. 5’-UTR, 5′ untranslated region. (B) Levels of GFP expression as a result of the location of the ACA site upstream from the AUG initiation codon. E. coli strain MG1655 was separately transformed with plasmid pUH-C carrying five different *gfp* reporter genes with an ACA site upstream from the initiation codon, ATG. Here we show a quantitative comparison of levels of GFP expression in samples induced by nalidixic acid (green bars) and in uninduced samples (blue bars). These data were calculated as percentages of fluorescence units. For each assay, 100% represents the results for the untreated sample.

To test whether the expression of a stress-induced MazF-mediated protein is a function of the number of nucleotides between the AUG initiator and the ACA MazF cleavage site, we used a green fluorescent protein (GFP) reporter system (Fig. 2A). In previous work (13), we constructed a similar GFP reporter system in which we demonstrated that *mazF*-mediated expression depends on the existence of an ACA MazF cleavage site upstream from the AUG initiator codon, permitting the generation of a MazF-processed mRNA that is translated by the STM. Here, we studied whether the distance of the ACA site from the AUG initiator plays such a crucial role in the expression of the stress-induced GFP reporter. The results are shown in Fig. 2B, where NA-induced samples are shown in green and the uninduced are shown in blue. As a control, we used this GFP reporter but without any ACA sites upstream from the AUG initiator (Fig. 2B, blue bars). As expected, under stressful conditions, which were obtained by the application of NA, a decrease in GFP expression was observed (Fig. 2B, the green bars on the left), probably due to the inability of the reporter to be efficiently translated by the STM system. In contrast, the presence of an ACA site up to 80 nucleotides upstream of the AUG initiator permitted an increase in GFP expression following induction with NA (Fig. 2B, compare green bars to blue bars). In contrast, when we inserted an ACA site at 20, 40, or 80 nucleotides upstream from the AUG initiator, induction by NA led to an increase in GFP expression (green bars). On the other hand, a decrease in GFP expression was found when the ACA sites were located at a distance of ≥100 nucleotides upstream of the AUG initiator (Fig. 2B), indicating that the existence of an ACA site within 80 to 100 nucleotide upstream of the AUG initiator is important for the generation of a MazF-processed mRNA and thereby for being preferentially translated by the STM system. These results corroborate our findings that most (85%) of the stress-induced MazF-mediated proteins described here (Table 1) were specified by mRNAs in which there were ACA sites within 93 nucleotides upstream from the AUG initiator.

## DISCUSSION

Here, we report our proteomic analysis of the entire group of E. coli proteins that are stress induced and MazF mediated (Table 1). That the proteins described really are MazF mediated is supported by two lines of evidence. (i) We compared results for E. coli strain MC4100 *relA*^+^ and for its deletion derivative MC4100 *relA*^+^ Δ*mazEF*, and proteomics was carried out on proteins whose increment of expression by the stress-induced conditions (application of NA) was above 1.2. Levels of expression vary between 1.28 and 9.96 and were an average of results from three independent experiments. (ii) About 85% of these proteins (Table 1) were found to be programmed by mRNAs carrying the ACA MazF cleavage site located at least 100 nucleotides upstream from the AUG initiation codon. These results corroborated with previous findings showing that in E. coli, under stressful conditions, MazF induction leads to the generation of the new translation machinery, STM, translating mRNAs carrying the MazF cleavage site ACA up to 100 nucleotides upstream of their AUG initiation codons (6). Still, here we found that about 15% of the stress-induced MazF-mediated proteins were translated from mRNAs without an ACA codon within 100 nucleotides upstream from the AUG initiation codon (Table 1); instead, they were translated from mRNAs carrying either an ACG or an ACU site. ACG and ACU are cleavage sites for the toxin ChpBK of the *chpBIK* TA system (14). Just as stressful conditions induce MazF activity, it is possible that similar stressful conditions induce *chpBIK* to generate ChpBK. ChpBK cleaving the mRNA molecule at the upstream ACG or ACU site would result in translation by the STM. Alternatively, the already stress-induced MazF might induce ChpBK synthesis, leading to cleavage upstream at ACG or ACU, again leading to translation by the STM.

In conclusion, most of the MazF-induced proteins characterized here contain an ACA motif within 100 nucleotides upstream of the AUG initiator (except for the few that use ACG or ACU). Thus, as previously reported (5–7), the upstream ACA motif is important for the translation by the stress-induced MazF-mediated machinery (STM). However, our data do not rule out the possibility of additional recognition motifs, and we are currently pursuing these.

In addition, many of the proteins characterized here have previously been described as stress-induced proteins. These include a group of chaperon-related proteins that participate in the prevention of protein unfolding occurring under stress. This category includes proteins ClpB (15), DnaK (16), GroL and GroS (17), and GrpE (18). The stress-induced proteins also include three proteases: (i) Lon, which is required for cellular homeostasis and for survival from DNA damage and developmental changes induced by stress (19); (ii) ClpX, which specifies components of the Clp protease complex (20); and (iii) FtsH, an ATP-dependent metalloprotease (21). The stress-induced proteins also include one heat shock protein, IbpA (22), proteins involved in carbohydrate metabolism, like GalU (23) and phosphoenolpyruvate carboxylase PckA (24), and three osmotic proteins, OsmC (25), OsmE (26), and OsmY (27). Another key protein is the DNA-binding protein from starved cells (Dps), which was shown to be involved in protection from multiple stresses (28).

However, it may be that the most interesting proteins that were obtained in our studies are those that are self-evidently also produced under regular (unstressed) growth conditions. In this category we can include (i) the well-known RNA polymerase sigma factor RpoD, which promotes the attachment of RNA polymerase to initiation sites (29, 30); (ii) translation initiation factor IF-3 (directed by *infC*), one of the essential components for the initiation of protein synthesis (31); (iii) transcription termination factor Rho (32); (iv) the 50S ribosomal proteins RplU and RpmA (33); and (v) 30S ribosomal protein Sra (34). These proteins, which are so central to bacterial survival, are synthesized under normal unstressed conditions by the canonical translation system. Under stressful conditions, however, when their mRNAs are processed by MazF at an ACA codon located upstream from its initiator, translation can take place by the STM system. Thus, we suggest that the location of the ACA site upstream of the AUG initiator may allow the protein to be synthesized both under stress conditions by STM and under normal conditions by the canonical translation machinery. Such dual-translation mechanisms enable the cells to prepare proteins for immediate functions while coming back from stressful conditions to normal growth conditions.

## MATERIALS AND METHODS

### Bacterial strains and plasmids.

We used E. coli MC4100 *relA*^+^ and its Δ*mazEF* derivative MC4100 *relA*^+^ Δ*mazEF* (35), E. coli MG1655 (13), and plasmid pUH-C (13).

### Characterizing the E. coli proteins induced by MazF under stressful conditions.

Early-logarithmic cells of either E. coli MC4100 *relA*^+^ or MC4100 *relA*^+^ Δ*mazEF* were grown in M9 minimal medium (without lysine and arginine) at 37°C until an optical density at 600 nm (OD_600_) of 0.5 was reached. Stressful conditions were generated by adding 100 µg/ml of nalidixic acid (NA) for 10 min, after which heavy arginine (_15_N^4^) and heavy lysine (_15_N^2^) were added at a concentration of 100 µg/ml to each of the cultures, which were further incubated without shaking for 5 min. Samples of these treated cultures were centrifuged, frozen in liquid nitrogen, and sent to the Smoler Protein Research Center in Haifa, Israel, for proteomic analysis.

### Experimental design and statistical rationale.

This study consists of proteome analyses of two group types, E. coli MC4100 *relA*^+^ and MC4100 *relA*^+^Δ*mazEF*, to discover newly synthetized proteins by pulsed SILAC. Triplicate samples from each group were examined, with each replicate pair being examined on different dates. Liquid chromatography-tandem mass spectrometry (LC-MS/MS) data from all 3 experiments were combined and analyzed. Peptide and protein‐level false discovery rates (FDRs) were filtered to 1% by using the target decoy strategy for identification. H/L ratios for all peptides belonging to a particular protein species were pooled; a ratio for each protein in each sample was calculated separately. As the purpose of the study was to identify the new proteins rather than to calculate the turnover of the proteins, no complex statistical test was used and no logarithmic transformation was done.

More-detailed information is provided in the following sections.

### Proteolysis and mass spectrometry analysis.

The proteins were extracted from the cell pellets in 9 M urea, 400 mM ammonium bicarbonate, 10 mM dithiothreitol (DTT), with 2 cycles of sonication. Ten micrograms of protein from each sample was reduced (60°C for 30 min), modified with 35 mM iodoacetamide in 400 mM ammonium bicarbonate (in the dark at room temperature for 30 min), and digested in 2 M urea, 80 mM ammonium bicarbonate with modified trypsin (Promega) at a 1:50 enzyme-to-substrate ratio overnight at 37°C. An additional second trypsinization was done for 4 h after diluting the urea concentration to 1 M.

The resulting tryptic peptides were desalted using C_18_ tips (Harvard) dried and resuspended in 0.1% formic acid. They were analyzed by LC‐MS/MS using a Q Exactive Plus mass spectrometer (Thermo) fitted with a capillary high-performance liquid chromatograph (HPLC; easy nLC 1000; Thermo). The peptides were loaded onto a homemade capillary column (25-cm, 75-μm internal diameter) packed with Reprosil C_18_‐Aqua (Dr. Maisch GmbH, Ammerbuch, Germany) in solvent A (0.1% formic acid in water). The peptide mixture was resolved with a (5 to 28%) linear gradient of solvent B (95% acetonitrile with 0.1% formic acid) for 105 min, followed by a 15-min gradient of 28 to 95% and 15 min at 95% acetonitrile with 0.1% formic acid in water at flow rates of 0.15 μl/min. Mass spectrometry was performed in a positive mode (*m/z* 350 to 1,800; resolution, 70,000) using a repetitively full MS scan followed by collision‐induced dissociation (high-energy collision dissociation [HCD], at a normalized collision energy of 35) of the 10 most dominant ions (> 1 charges) selected from the first MS scan. The AGC settings were 3 × 10^6^ for the full MS scan and 1 × 10^5^ for the MS/MS scans. The intensity threshold for triggering MS/MS analysis was 1 × 10^4^. A dynamic exclusion list was enabled with an exclusion duration of 20 s.

The mass spectrometry data of all 3 repeats were analyzed using MaxQuant software v.1.5.2.8. (36) for peak picking identification and quantitation using the Andromeda search engine, which searches for tryptic peptides against the *Escherichia coli* K-12 UniProt database (4,459 entries), with mass tolerances of 20 ppm for the precursor masses and 20 ppm for the fragment ions. Oxidation on methionine and protein N terminus acetylation were accepted as variable modifications, and carbamidomethyl on cysteine was accepted as a static modification, as the percentage of the carbamylation was very low. Minimal peptide length was set to 6 amino acids, and a maximum of two miscleavages was allowed. Peptide‐ and protein‐level false discovery rates (FDRs) were filtered to 1% using the target decoy strategy. Protein tables were filtered to eliminate the identifications from the reverse database and common contaminants and single peptide identifications. The data were quantified by SILAC analysis using the same software. H/L ratios for all peptides belonging to a particular protein species were pooled, providing a ratio for each protein.

### Construction of GFP reporters for studying the effect of ACA sites upstream of the AUG initiator on GFP.

To construct the genes for our green fluorescent protein (GFP) reporters, we previously used a GFP variant derived from Emerald-GFP (EmGFP; pRSET-*em-gfp*; Invitrogen, Carlsbad, CA, USA). Emerald-GFP has a distinct excitation peak at 487 nm and a distinct emission peak at 509 nm. In this *gfp* gene, we changed all the ACA sites to ATA sites, thus eliminating all MazF cleavage sites while maintaining the protein coding sequence (13). Moreover, this GFP reporter gene carries no ACA sites upstream of the initiation codon ATG (up to 135 nucleotides). The *gfp* construct was cloned into a pUH-C plasmid (13). Here, we used this *gfp* construct as a platform to insert ACA sites upstream of the initiation codon ATG at 5 different locations. The ACA codons were inserted at different distances (numbers of nucleotides) upstream of the initiation codon ATG: –20, −40, −80, −100, and −120 nucleotides.

In order to create each ACA insertion, a forward primer (PF) and a reverse primer (PR) were designed. PCRs were carried out using the *gfp* construct carrying no ACA sites (on a pUH-C plasmid) as a template. To create newly mutated synthesized plasmids and to prevent extra mutations, we carried out the PCR program with only a few cycles of annealing: 5 cycles for the first annealing stage and 10 cycles for the second annealing stage. Finally, to eliminate the original plasmid and leave only newly mutated synthesized unmethylated plasmids, we added the enzyme DpnI to cut the methylated DNA, leaving the unmethylated DNA intact. We confirmed all of the generated mutations by sequencing.

We inserted ACA sites at each of five specific locations, 20, 40, 80, 100, and 120 nucleotides upstream from the ATG initiation codon, by using the primers in Table 2.

**TABLE 2:** Primers used

Primer	Sequence
P**F** ACA 20up	GGCGTATTTTG**ACA**CCTAACGAGG
P**R** ACA 20up	CCTCGTTAGG**TGT**CAAAATACGCC
P**F** ACA 40up	CTCGTTGGAG**ACA**TTCATGGCGT
P**R** ACA 40up	ACGCCATGAA**TGT**CTCCAACGAG
P**F** ACA 80up	ATATTGAGCAG**ACA**CCCCGGTGAAG
P**R** ACA 80up	CTTCACCGGGG**TGT**CTGCTCAATAT
P**F** ACA 100up	CGAAGATATCG**ACA**GAGTTAATAT
P**R** ACA 100up	ATATTAACTC**TGT**CGATATCTTCG
P**F** ACA 120up	GGGGTGCTCG**ACA**TAAGCCGAAG
P**R** ACA 120up	CTTCGGCTTA**TGT**CGAGCACCCC

### Growth conditions and assays for measuring GFP expression.

We used plasmids bearing each of the *gfp* reporter genes to transform E. coli MG1655 (WT). We grew the transformed bacteria in 10 ml M9 medium containing 0.2% glucose and 100 μg/ml ampicillin at 37°C with shaking (250 rpm) until the cultures reached an OD_600_ of 0.4 to 0.5. Using black 96-well plates, we applied triplicate samples to wells, not treating the controls and treating the experimental samples with 100 μg/ml of NA to induce MazF activity. We detected GFP levels using a FLUOstar spectrophotometer. Using a 485 ± 15 nm excitation filter and a 520 ± 15 nm emission filter over a total time of 750 min, we measured fluorescence 150 times at intervals of 300 s (total time of experiment, 750 min). We maintained the temperature in the device at 37°C. The GFP fluorophore was excited with 1,000-CW lamp energy, and the fluorescence in each well was measured for 5 s (FLUOstar galaxy; BMG Labtech).

### Data availability.

The mass spectrometry proteomics data have been deposited in the ProteomeXchange Consortium (http://proteomecentral.proteomexchange.org) via the PRIDE partner repository with the data set identifier PXD010101.

10.1128/mBio.00340-19.2 TABLE S1 Protein identification and quantification. The protein group output from MaxQuant software is shown. The table also reports on protein H/L ratios. Each row corresponds to the protein group. Whenever the set of identified peptides for one protein was identical to or completely contained in that of another protein, both proteins were joined in the same protein group by MaxQuant. Download Table S1, XLSX file, 0.8 MB. Copyright © 2019 Nigam et al. 2019 Nigam et al. This content is distributed under the terms of the Creative Commons Attribution 4.0 International license.

10.1128/mBio.00340-19.3 TABLE S2 Peptide identification and quantification from the output table from MaxQuant software. For both tables, “Protein IDs” are identifiers of proteins contained in the protein group (multiple entries that could not be differentiated on the basis of peptide evidence). They are sorted by number of identified peptides in descending order. “Protein names” are names of proteins. “Gene names” are names of genes associated with the proteins. “Sequence coverage [%]” is the percentage of the sequence that is covered by the identified peptides of the best protein sequence contained in the group. “Mol. weight [kDa]” is the molecular weight of the leading protein sequence contained in the protein group. “Sequence length” is the length of the leading protein sequence contained in the group. “PEP” is the posterior error probability of the identification. This value essentially operates as a *P* value, where smaller is more significant. “Number of proteins” signifies the number of proteins contained within a group. This corresponds to the number of entries in the column “Protein IDs.” “Razor + unique peptides” is the total number of razor plus unique peptides associated with the protein group (i.e., these peptides are shared with another protein group). Here, distinct peptide sequences are counted. Modified forms or different charges are counted as one peptide. “2 Unique peptides” is the total number of unique peptides associated with a protein group (i.e., these peptides are not shared with another protein group). “Intensity” is the summed-up extracted ion current (XIC) of all isotopic clusters associated with the peptide sequence, and protein intensities sum the intensities of all peptides assigned to the protein group. “Ratio H/L” is the ratio between two heavy- and light-label partners. In cases in which the ratio was not determined and both H and L intensities were available, the ratio was calculated and is printed in italics. “Ratio H/L variability [%]” is the coefficient of variability over all redundant quantifiable peptides. It is calculated as the standard deviation of the naturally logarithmized ratios times 100. “Ratio H/L count” is the number of redundant peptides (MS1 features) used for quantitation. Download Table S2, XLSX file, 6.6 MB. Copyright © 2019 Nigam et al. 2019 Nigam et al. This content is distributed under the terms of the Creative Commons Attribution 4.0 International license.
