# Supplementary figures and images for: Stress-Induced MazF-Mediated Proteins in Escherichia coli
Source: mBio. 2019 Mar 26;10(2):e00340-19. doi: 10.1128/mBio.00340-19 (PMC6437054; doi:10.1128/mBio.00340-19)

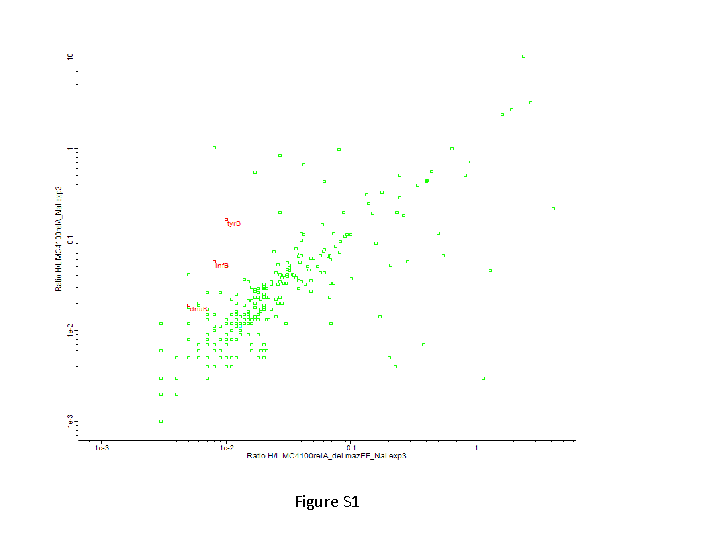

Supplement: FIG S1 [file mBio.00340-19-sf001.tif]
